# Explosive detection canines in the field: a multi-site black box validation study

**DOI:** 10.3389/fvets.2025.1668317

**Published:** 2025-10-16

**Authors:** Michelle Karpinsky, Haylie Browning, Adele Quigley-McBride, Paul Bunker, Will Chapman, Paola A. Prada-Tiedemann, Lauryn E. DeGreeff

**Affiliations:** ^1^Department of Chemistry and Biochemistry, Global Forensic and Justice Center, Florida International University, Miami, FL, United States; ^2^Department of Environmental Toxicology, Texas Tech University, Lubbock, TX, United States; ^3^Department of Psychology, Simon Fraser University, Burnaby, BC, Canada; ^4^Chiron K9, Somerset, TX, United States; ^5^Department of Forensic Science, Noblis, Reston, VA, United States

**Keywords:** standardization, explosives, explosive detection canines, black box study, validation

## Abstract

In 2009, the National Research Council called upon the forensic science community to standardize the best practices and guidelines in the collection and analysis of evidence with the goal of ensuring quality and consistency within the field. In response to this need, the Organization of Scientific Area Committees for Forensic Science (OSAC) was established to coordinate the development of best practices and standards in the forensic sciences. The OSAC Dogs and Sensors subcommittee was part of this initiative focusing on standardizing training and certification protocols for canine detection teams. Though efforts to create and promote such standards are ongoing worldwide, the developed assessments for both training and operational contexts have yet to be empirically validated. As a first step toward addressing this gap, a proof-of-concept black box study was carried out to assess the OSAC explosive canine detection standard based on performance of explosive detection canines. The evaluations were held in three separate geographic locations with a total of 56 canine/handler teams, took place over 2 days, and included searches recommended within the ANSI/ASB Standard 092 as well as scenarios designed to more closely mimic what the teams might experience in practice. Overall, the results from the individual canine/handler team responses revealed that no team would have passed the OSAC certification; however, the results indicated comparable performance on both assessment types (standard assessments and operational scenarios). Additionally, canine/handler performance varied significantly across all three trials in both correct alert, false alert rates, and detection success rate across the mandatory six different explosive types presented. These findings suggest that the performance on Standard 092 certification assessments may predict operational effectiveness. The results also suggest that the variation in performance is attributable to the diversity of training aid material routinely available to the participating teams.

## Graphical abstract

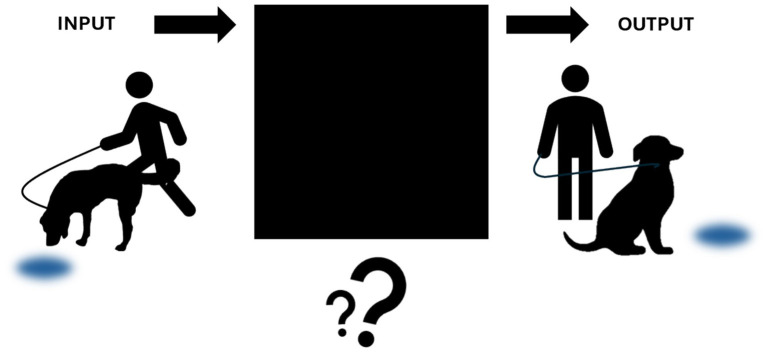

## 1 Introduction

According to the Organization of Scientific Area Committees for Forensic Science (OSAC), forensic science is a multidisciplinary field categorized into seven main Subject Area Committees (SACs): biology, seized drugs and toxicology, trace evidence, physics/pattern interpretation, scene examination, medicine, and digital/ multimedia. Detection canines serve as an investigative tool in criminal investigations, hence their status as a forensic discipline and the inclusion of the Dogs and Sensors subcommittee under the scene examination SAC. Canines are considered a biological sensor and are extensively utilized by police and military forces to identify substances such as drugs, explosives, and human remains. Dogs have a highly developed olfactory system, possessing nearly 300 million nasal olfactory receptors, superior sensitivity, measured as low as parts-per-trillion (ppt), and selectivity rivaling other field detection technologies (1–5). Because of this, though other highly sophisticated analytical instruments are available for trace detection, canine detection remains one of the most widely utilized and effective technologies available for field detection of explosive threats (6). Nevertheless, despite their impressive detection capabilities, methods for assessing their performance are limited and have not yet been scientifically validated (5, 7).

Efforts to standardize forensic practices in the United States, including the use of canines for detection, gained momentum in the early 1990s, when the FBI sponsored the development of the Scientific Working Groups (SWGs) to improve consistency and promote best practices across forensic disciplines (8). There were approximately 22 SWGs formed, each dedicated to a specific area of specialization such as DNA analysis, bloodstain pattern analysis, seized drugs, and friction ridge analysis. Among these was the Scientific Working Group on Dog and Orthogonal Detection Guidelines (SWGDOG), established in 2004 to develop best practice guidelines for canine detection. SWGDOG’s main objective was to improve the performance, reliability, and courtroom defensibility of canine/handler teams. Between 2004 and 2014, SWGDOG published 24 guidelines encompassing more than 400 pages of recommendations and resources (9).

Further, in 2008, a document titled *Wrongful convictions and forensic science: The need to regulate crime labs* by P. C. Giannelli drew attention to the failures of crime labs and the lack of standardization within forensic science (10). This document called for standardization, certification, and accreditation throughout all disciplines. To rectify this, in 2009, the National Research Council published *Strengthening Forensic Science in the United States: A Path Forward*, scrutinizing the current state of forensic science in the US. The report highlighted the lack of standardization within disciplines, permitting substantial variability in how evidence was collected, analyzed, and translated into forensically-relevant results (11).

Many forensic techniques, including canine detection, relied on practices passed down through informal training rather than validated, consensus-based methods. Thus, in response, the National Institute of Science and Technology (NIST) created the Organization of Scientific Area Committees (OSAC) for Forensic Science in 2014 to integrate and centralize the development of best practice recommendations and standards under a single organization rather than convening individual SWGs. The Dogs and Sensors subcommittee was created within Scene Examination Scientific Area Committee to take on the work previously done by SWGDOG. Existing SWGDOG guidelines were revised to meet OSAC criteria so that they could be considered by a Standards Development Organization (SDO) and placed on the OSAC registry (12).

In December of 2022, the OSAC Dogs and Sensors subcommittee published ANSI/ASB Standard 092 titled *Standard for Training and Certification of Canine Detection of Explosives*. This standard was approved by the American National Standards Institute (ANSI) and American Standards Board (ASB) of the American Academy of Forensic Science (AAFS). The document (hereafter, *Standard 092*) outlines baseline protocols for training and certifying explosive detection canines, including information such as the minimum requirements, best practices, standard protocols, and terminology (13). The goal of Standard 092 is to promote consistency and operational effectiveness across explosive detection dog (EDD) teams by standardizing certification testing to ensure all certified teams meet the same, expert-defined criteria. Additionally, for a discipline such as canine detection, where training and assessment are often based on personal experience or incomplete descriptions of requirements, producing a standard ensures that operational teams are trained under a similar process to promote a baseline for quality control measures and accountability (14).

In theory, the idea of a standard training and certification protocol created by experts within the field would provide uniformity throughout all operational units; however, without observing these standards in practice, it is difficult to determine the practicality, as well as the relevancy and efficacy of the standards being developed. To know whether meeting the Standard 092 certification criteria means a team is ready for real-world scenarios, the standard needs to be empirically evaluated to determine whether teams that meet or exceed the criteria in the standard also perform at a high level in an operational context. To assess the effectiveness of ANSI/ASB Standard 092 for predicting real-world performance, a proof-of-concept black box study was developed.

Black box studies aim to provide a quantifiable snapshot of the efficacy of forensic techniques, without seeking to understand how successful or unsuccessful performance comes about. These studies have become increasingly common since the 2009 NRC report, particularly to evaluate disciplines that involve subjective elements, such as pattern-matching techniques (15–20). For example, the first large-scale study to measure the accuracy and reliability of latent print examiners’ decisions about the matching of approximately 100 pairs of latent and exemplar prints was reported in 2011 (19). Subsequent studies examined other pattern-matching disciplines, including bloodstain pattern analysis (16), shoeprint examination (18), handwriting comparison (17), and DNA mixture interpretation (15). These efforts have highlighted the limitations of existing practices in these fields while also providing empirical evidence to inform error rate estimates that can be used to support courtroom testimony.

In the study herein, the authors apply the paradigm of the black box study to canine detection. Because both the canine and the handler are active participants in the detection process and coordinate to complete their assigned task, the discipline is unique among the forensic sciences. One unique aspect is that there are more points at which a judgment can lead to an error. The canine may fail to detect an explosive (a “miss” or a “false negative”) or may alert when no explosive is present (“false alert” or “false positive”). In some cases, the canine responds correctly, but the handler misinterprets or fails to interpret the alert, also resulting in a false positive or negative. Ultimately, it is the handler’s call that determines the outcome in practice because they serve as the conduit through which the canine’s detection is communicated.

Like other forensic disciplines, training practices for canine/handler teams can vary widely. Regardless of the specific regimen, the primary goal is to maximize true positives while minimizing false negatives and false positives. Most forensic disciplines also require practitioners to demonstrate competency through proficiency testing under controlled conditions before working on real-world cases. In canine detection, however, certification tests can differ substantially between agencies and organizations—even within agencies and organizations—so the field lacks a uniform approach or criterion for certification.

The implementation and validation of standards, such as ANSI/ASB Standard 092, within the forensic canine detection community provides a framework for demonstrating the accuracy and reproducibility of canine/handler team performance, as well as evaluating the efficacy and practical applicability of the standard itself. The goal of the study was to utilize the black box framework to objectively assess the OSAC explosive detection standards, and more broadly, the performance of EDD teams in the United States. This was achieved through operational canine assessments held in three different locations across the United States. The assessment consisted of two components. The first adhered to the certification testing framework outlined in OSAC National Registry Standard 092, *Standard for Training and Certification of Canine Detection of Explosives* (ANSI/ASB Standard 092). The second compared EDD team performance on the prescribed Standard 092 certification assessment with their performance in a second set of assessments involving more realistic and operationally challenging explosive detection scenarios.

## 2 Methods

All the protocols within this study were reviewed and approved by the Texas Tech Institutional Animal Care and Use Committee (IACUC, Protocol 2023–1,398) as well as the Florida International University IACUC (Protocol #201805). The study consisted of three trials conducted within the Southwestern (SW), Southeastern (SE), and Western (W) regions of the United States. Table 1 provides information about the trial locations, dates the trials were held, temperature range of the duration of the outdoor and vehicle searches, the humidity, and total number of canines that participated on that day of the trial. All indoor searches were held at room temperature.

**Table 1:** Trial number, location of each trial, dates of the first and second day of each trial, temperature for all searches held outdoors, humidity, and the total number of dogs on Day 1 and 2.

Trial #	Location	Date	Temperature	Humidity	Total # of dogs
1	SW	7/30/2024	90.0–93.9°F	61%	20
		7/31/2024	91.0–96.1°F	52%	15
2	SE	1/19/2025	73.0–81.0°F	65%	13
		1/20/2025	60.1–63.0°F	80%	15
3	W	5/21/2025	57.9–69.1°F	31%	19
		5/22/2025	57.0–64.9°F	37%	19

### 2.1 Canine/handler team information

A total of 56 canine/handler teams from law enforcement, government, and private companies participated in this study; however, not all teams participated in all areas of the study (See Supplementary information). Prior to the trial, participating canine teams were asked to provide information about their canine (breed, age, number of years the dog has been in service) and themselves (number of years the handler has been in service), and the most recent year of successful certification (see Supplementary information for these details). To maintain anonymity, canine/handler teams were assigned team numbers that cannot be traced back to the original team or their organization. The participating canines were all operational dogs that were trained, housed, and handled by their handler or agency.

### 2.2 Materials

The six explosives used in the study were those dictated as “required” by Standard 092. For security reasons, these will be referred to as Explosives 1 through 6. The explosives were purchased from OMNI explosives. According to Standard 092, the minimum amount of each explosive for the certification assessments should be no less than ¼ lbs. (113.5 g) or 8 ft. in length of 50 gr/ft. (9 g/m), and thus this quantity was utilized in all searches related to the certification assessment. For non-operational odor recognition assessments, such as the odor recognition test (ORT), a maximum of ¼ lbs. (113.5 g) or 8 ft. (metric) in length of 50 gr/ft. (9 g/m) of explosive material was used. These quantity specifications were used in all certification trials. For the “real-world” scenarios, the amounts of explosives used are listed below in Tables 2, 3.

**Table 2:** Day 1 searches of the trial, including the type of search, the requirements needed for the search, the targets, distractors, and blanks placed for the search, the container the samples were presented in, and the amount of targets placed out.

Day 1				
	Sample	Type	Container	Mass of explosive(s)
Morning				
Scenario 1 - People with Baggage				
Explosive 2 and Explosive 5		Target	Pressure Cooker in Baggage	Residue (<1 g) and 0.61 m
Masking tape		Distractor	Pressure Cooker in Baggage	N/A
Empty baggage × 8		Blank	Baggage	N/A
Scenario 2 - Pile of boxes				
Blank boxes		Blank	Box	N/A
Eucalyptus and rosemary spray		Distractor	Box	N/A
Scenario 3 - Outdoor search				
Explosive 3 w/Tang		Target	MODD	28 g
Tang		Distractor	MODD	N/A
Eucalyptus and rosemary spray		Distractor	TADD	N/A
Fragrance spray		Distractor	TADD	N/A
Empty MODD		Blank	MODD	N/A
Standard 092 - Vehicle search				
Explosive 1		Target	TADD	115 g
Sharpie		Distractor	TADD	N/A
Conditioner		Distractor	TADD	N/A
Rubber bands		Distractor	TADD	N/A
Empty TADD × 2		Blank	TADD	N/A
Cars x4		Blank	N/A	N/A
Afternoon				
Standard 092 - Room Search 1				
Explosive 3		Target	TADD	115 g
Crayons		Distractor	TADD	N/A
Steak seasoning		Distractor	TADD	N/A
Shampoo		Distractor	TADD	N/A
Empty TADD		Blank	TADD	N/A
Standard 092 - Room Search 2				
Ground cinnamon		Distractor	TADD	N/A
Liquid glue		Distractor	TADD	N/A
Masking tape		Distractor	TADD	N/A
Empty TADD x2		Blank	TADD	N/A
Standard 092 - Room Search 3				
Explosive 2		Target	TADD	115 g
Glue stick		Distractor	TADD	N/A
Air freshener		Distractor	TADD	N/A
Packing peanuts/packing wrap		Distractor	TADD	N/A
Empty TADD		Blank	TADD	N/A
Standard 092 - Parcel Search				
Explosive 5		Target	TADD in box	2.44 m
Explosive 4		Target	TADD in box	115 g
Coffee grounds		Distractor	TADD in box	N/A
IPA wipes		Distractor	TADD in box	N/A
Nitrile gloves		Distractor	TADD in box	N/A
Boxes × 5		Blank	N/A	N/A
Standard 092 - ORT 1				
Explosive 1		Target	TADD	50 g
Explosive 2		Target	TADD	50 g
Explosive 3		Target	TADD	50 g
Explosive 4		Target	TADD	50 g
Explosive 5		Target	TADD	1.22 m
Explosive 6		Target	TADD	50 g
Play-Doh		Distractor	TADD	N/A
Tang		Distractor	TADD	N/A
Motor oil		Distractor	TADD	N/A
Anti-static bag		Distractor	TADD	N/A
Taco seasoning		Distractor	TADD	N/A
Latex gloves		Distractor	TADD	N/A
Empty TADDs × 6		Blank	TADD	N/A
Empty boxes		Blank	N/A	N/A

**Table 3:** Day 2 searches of the trial, including the type of search, the requirements needed for the search, the targets, distractors, and blanks placed for the search, the container the samples were presented in, and the amount of targets placed out.

Day 2				
	Sample	Type	Container	Mass of explosive(s)
Morning				
Scenario 1 - Interior Vehicle Search				
Explosive 4 and Explosive 5		Target	Plastic Wrap	115 g and 0.61 m
Play-Doh in plastic wrap		Distractor	Plastic Wrap	N/A
Plastic wrap		Blank	Plastic Wrap	N/A
Scenario 2 - Piles of luggage				
Explosive 3 w/sugar		Target	MODD	28 g
Sugar		Distractor	MODD	N/A
Used (latex + nitrile) gloves		Distractor	Luggage	N/A
Empty MODD		Blank	MODD	N/A
Scenario 3 - Outdoor Search				
Explosive 6		Target	Paper bag	115 g
Panda express leftovers		Distractor	Plastic bag	N/A
Nerds + rocks		Distractor	Paper bag	N/A
Dryer sheets		Distractor	N/A	N/A
Empty paper bag w/rocks		Blank	Paper bag	N/A
Standard 092 - ORT 2				
Explosive 1		Target	TADD	50 g
Explosive 2		Target	TADD	50 g
Explosive 3		Target	TADD	50 g
Explosive 4		Target	TADD	50 g
Explosive 5		Target	TADD	1.22 m
Explosive 6		Target	TADD	50 g
Play-Doh		Distractor	TADD	N/A
Tang		Distractor	TADD	N/A
Motor oil		Distractor	TADD	N/A
Anti-static bag		Distractor	TADD	N/A
Taco seasoning		Distractor	TADD	N/A
Latex gloves		Distractor	TADD	N/A
Empty boxes		Blank	N/A	N/A
Afternoon				
Standard 092 - Room Search 1				
Explosive 3		Target	TADD	115 g
Steak seasoning		Distractor	TADD	N/A
Conditioner		Distractor	TADD	N/A
Coffee grounds		Distractor	TADD	N/A
Empty TADD		Blank	TADD	N/A
Standard 092 - Room Search 2				
Explosive 5		Target	TADD	2.44 m
Isopropanol wipes		Distractor	TADD	N/A
Rubber bands		Distractor	TADD	N/A
Air freshener		Distractor	TADD	N/A
Empty TADD		Blank	TADD	N/A
Standard 092 - Room Search 3				
Elmers glue		Distractor	TADD	N/A
Sharpie		Distractor	TADD	N/A
Masking tape		Distractor	TADD	N/A
Empty TADD × 2		Blank	TADD	N/A
Standard 092 - Room Search 4				
Explosive 1		Target	TADD	115 g
Nitrile gloves		Distractor	TADD	N/A
Packing peanuts/packing wrap		Distractor	TADD	N/A
Crayons		Distractor	TADD	N/A
Empty TADD		Blank	TADD	N/A
Standard 092 - Parcel Search				
Explosive 2		Target	TADD in box	115 g
Explosive 4		Target	TADD in box	115 g
Ground cinnamon		Distractor	TADD in box	N/A
Shampoo		Distractor	TADD in box	N/A
Glue stick		Distractor	TADD in box	N/A
Boxes × 5		Blank	N/A	N/A
Standard 092 - Baggage Search				
Banana		Distractor	TADD in Luggage	N/A
Dryer sheets		Distractor	TADD in Luggage	N/A
Duct tape		Distractor	TADD in Luggage	N/A
Luggage × 7		Blank	N/A	N/A

Materials were handled in a specific way to minimize cross-contamination of odors. Explosives were weighed out one at a time into anti-static bags, placed into separate metal paint cans based on explosive type, and properly sealed. The area’s surface was decontaminated using an alcohol wipe and allowed to dry before the next explosive was prepared. Target odors were prepared a minimum of 18 h in advance of the study. Olfactory distractors for the trials were chosen from a variety of commonly used household items, non-target items used in the experiment, and other items thought to cause false alerts (refer to Tables 2, 3). The chosen distractors ranged from having low to minimal odor to being highly odorous. Distractors and blanks were housed separately from the explosives. Explosives and distractors were placed in 8 oz. Training Aid Delivery Devices (TADDs; SciK9) on all occasions, unless otherwise noted. A Mixed Odor Delivery Device (MODD) was used in the scenarios to safely deliver the odor of targets that would traditionally be detected as a mixture (21). For the ORT, all target odors, blanks, and distractor odors were presented in 4 × 4 × 6-inch or 6 × 6 × 6-inch boxes that were purchased from Uline.

### 2.3 Experimental set-up

The study consisted of three trials conducted within different regions of the United States (see Table 1). Testing sites used for each location were chosen based on availability and the standard-dictated space requirements. The SW trial took place in an office building, the SE trial at a university, and the W trials in a prison. Search areas varied across trial locations but were kept as consistent as logistically possible. The organization of when these searches took place remained consistent across all three trials. Each trial took place over 2 days and included searches in compliance with Standard 092 as well as more operationally realistic searches, referred to as “real-world scenarios.” Standard 092 included searches of rooms, parcels, vehicle exteriors, and luggage, as well as odor recognition tests. The ORT is a test of the canine’s olfactory ability to alert to target odor(s) in a controlled manner where the odor is readily available, but still visibly concealed from the canine/handler team. The targets odors are placed in a in line-up surrounded by distractor odors and blanks 3 ft. apart from each other. Scenarios were created to mimic searches canine/handler teams may encounter in real search operational contexts. Tables 2, 3 list all searches for each day of the trial, as well as the Standard 092 requirements for each search, the type of containment used, and the explosive and distractor odors presented. Table 4 provides the timing each team was permitted for each search, as well as the Standard 092 requirements for each search. A more detailed set-up and explanation of each search can be found in the Supplementary material.

**Table 4:** Search times allotted to teams throughout the trial.

Search type	Search parameters	Search time
Day 1		
Scenario 1 - People w/ Baggage	8 People w/baggage	3 min
Scenario 2 - Pile of Boxes	Pile of cardboard boxes	2 min
Scenario 3 - Outdoor Search	Minimum of 22,500 ft^2^	3 min
Standard 092 -Vehicle Search	10 Vehicles	20 min
Standard 092 - Room Searches	Between 200 ft^2^ and 1,200 ft^2^	3 min
Standard 092 - Parcel Search	10 Boxes, 3 ft. in between each	10 min
Standard 092 - ORT 1	24 Boxes, 3 ft. in between each	5 min
Day 2		
Scenario 1 - Interior Vehicle Search	3 Vehicles	6 min
Scenario 2 - Pile of Luggage	3 Piles of stacked luggage	3 min
Scenario 3 - Outdoor Search	Minimum of 22,500 ft^2^	3 min
Standard 092 - ORT 2	24 Boxes, 3 ft. in between each	5 min
Standard 092 - Room Searches	Between 200 ft^2^ and 1,200 ft^2^	3 min
Standard 092 - Parcel Search	10 Boxes, 3 ft. in between each	10 min
Standard 092 - Baggage Search	10 Luggage, 3 ft. in between each	10 min

All rooms utilized in each location had the minimum required space dimensions (Table 4) and included extra furniture and other items such as desks, cabinets, office supplies, etc. Target odors were placed within search areas a minimum of 30 min in advance of the first search for both the morning and afternoon sessions to allow for the odor to penetrate into the room (soak). Targets were left in place and were not moved in between canines. The searches conducted were single-blind, meaning the evaluators knew the placement and number of the targets, but the handlers did not. The search order of canine/handler teams was randomized prior to the start of each trial. Canine teams were not permitted to view the assessment area beforehand, nor were they permitted to watch any other canine team perform the assessments. Two evaluators were present for every search and provided instructions to the canine/handler teams about the search area and time limits before each search (Table 4). Handlers were instructed to provide a verbal indication if their canine alerted and to specify where the alert occurred. Evaluators then gave a verbal indication of whether the team was correct. The team continued to search until the target was found, the room was cleared, or the time limit was reached. All handlers were given the opportunity to allow their canines to search on or off leash.

### 2.4 Statistical analysis

Assessment sheets from each evaluator were collected and compared, and the canine responses were coded onto an Excel spreadsheet. The percent positive alert rates were calculated by the total number of positive alerts throughout the entirety of the trial divided by the total number of times the target odorant was present throughout the trial. The false alert rates were the number of total false alerts throughout the trial divided by the total amount of blanks and distractors present at each portion of the trial. Statistical analysis was completed using the Chi-square test (Microsoft Excel 365) to compare performance between trials as well as to compare the performance between “real-world” scenarios and Standard 092. The results were considered statistically significant if *p* ≤ 0.05. Additional multi-level logistic regression analyses are available in the Supplementary material that account for the variation attributable to canine/handler team and trial location, but these show the same effects as the more parsimonious Chi Square analyses. As a result, we have chosen to present the simpler analyses in the main body of the paper.

## 3 Results

### 3.1 Evaluation of the overall trials

**Figure 1:**
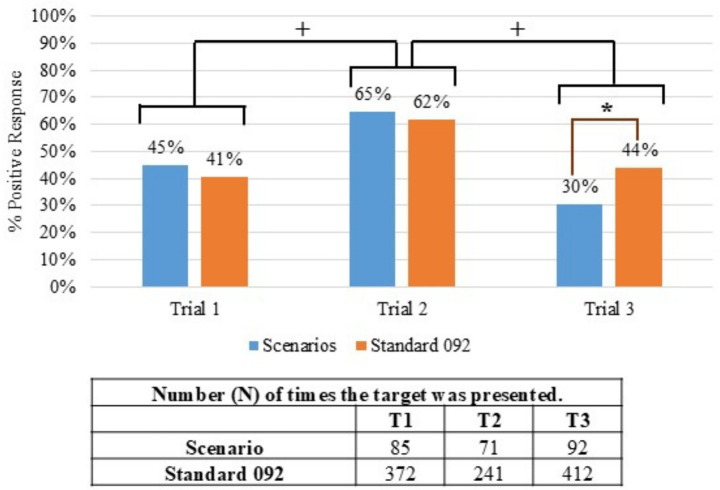
Percentage of positive alerts within the scenarios and Standard 092 across all three trials. (+) indicates a significant difference between trials, and the (*) indicates a significant difference between Standard 092 and Scenario within the same trial (*p* ≤ 0.05).

A total of 56 teams participated throughout the course of the study. Twenty (20) teams participated in Trial 1 (SW), 17 teams participated in Trial 2 (SE), and 19 teams participated in Trial 3 (W). Figure 1 provides the summarized results from the real-world scenarios and Standard 092 certification assessments for each trial. Canine/handler teams performed significantly better in Trial 2 than in either Trial 1 (χ^2^ [1, *N* = 27] = 33.16, *p* < 0.001) or Trial 3 (χ^2^ [1, *N* = 26] = 24.10, *p* < 0.001). Further comparison between scenarios and standards revealed that teams in Trial 3 performed significantly better on Standard 092 than in the scenario (χ^2^ [1, *N* = 19] = 5.64, *p* = 0.018) while in Trials 1 and 2, the teams depicted no significant difference in performance between Std 092 and the scenarios.

**Figure 2:**
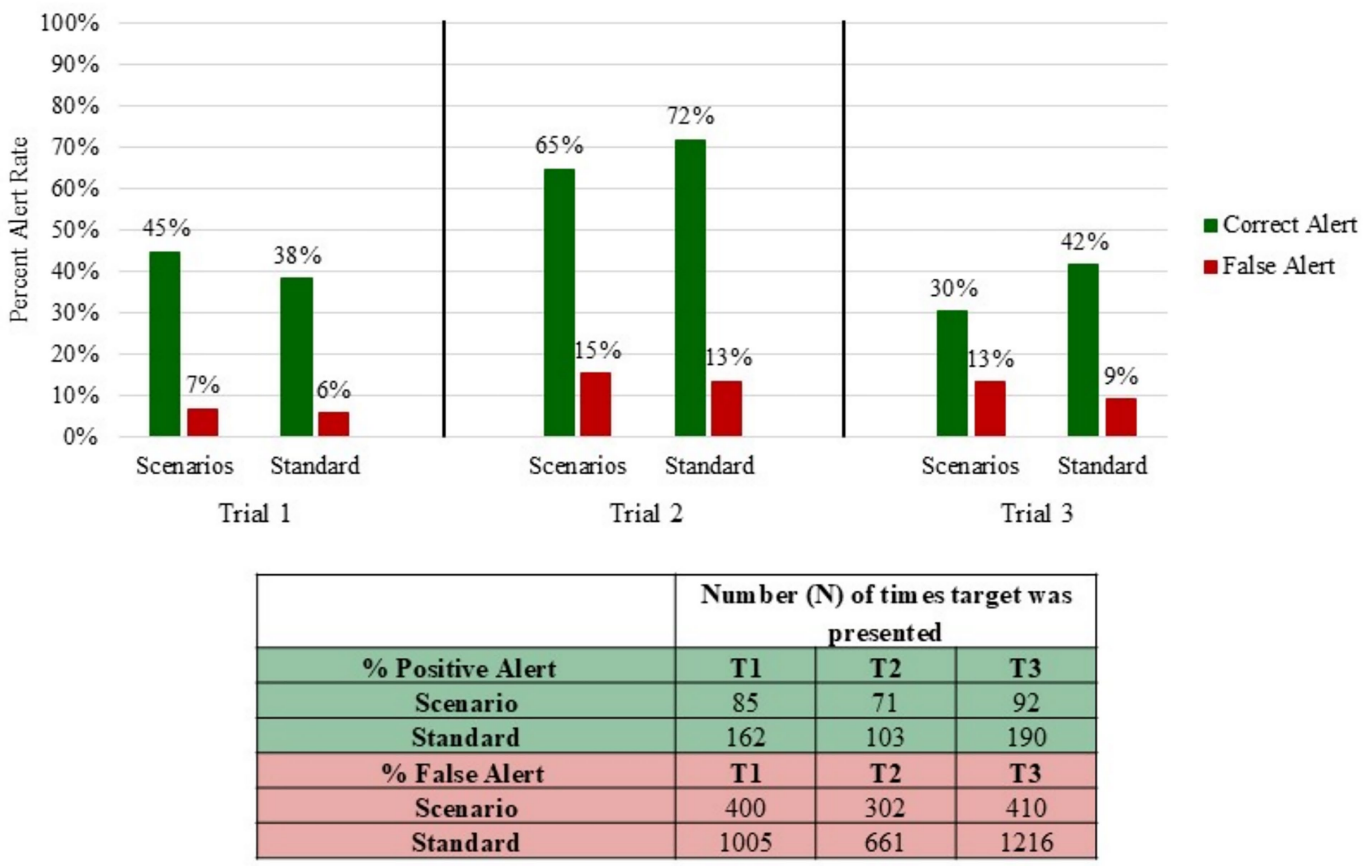
Percentage of positive and false alerts that occurred within the scenarios and the standard in the three trials. The ORT was excluded from the calculation of both positive and false alerts.

**Figure 3:**
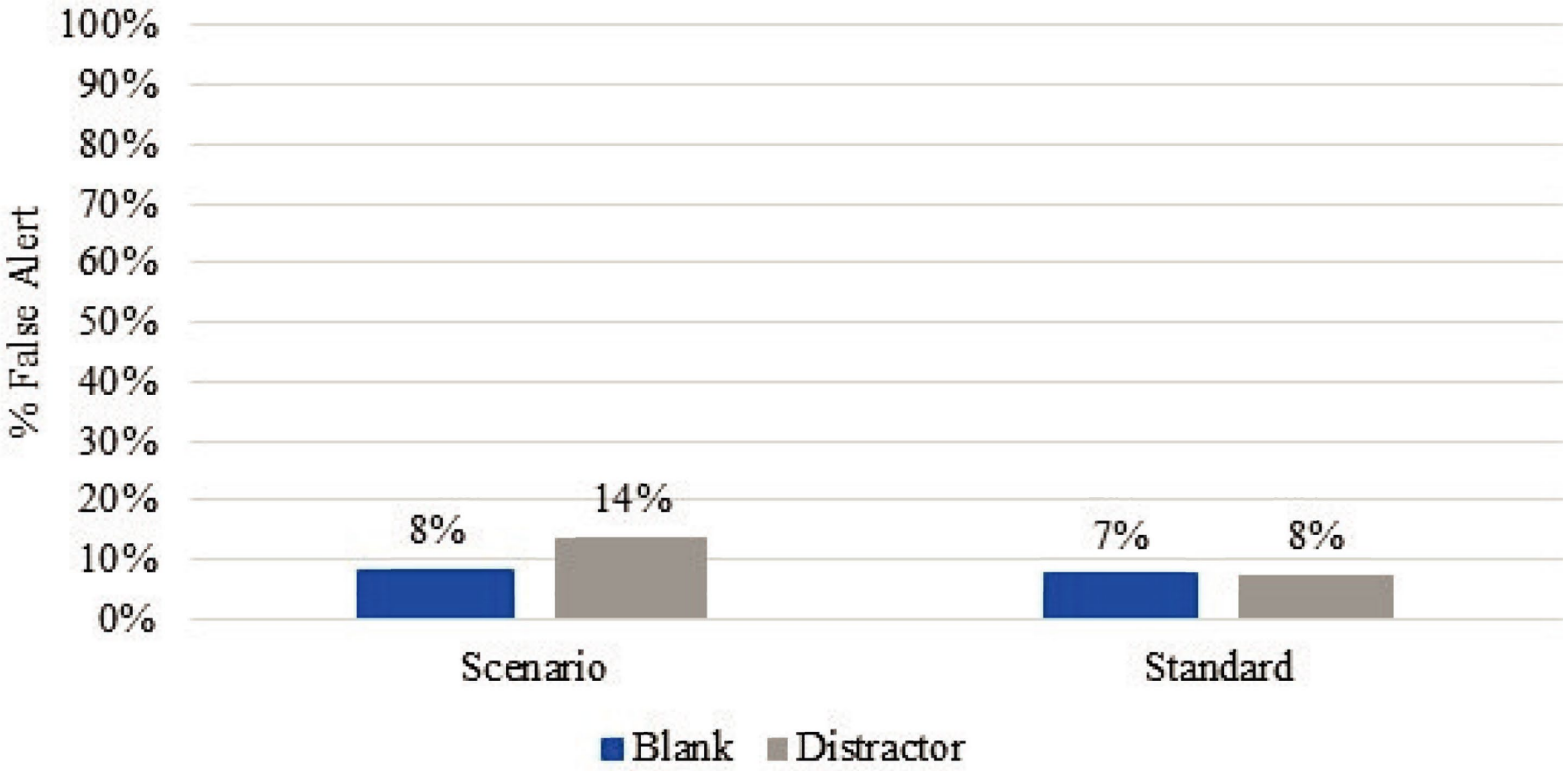
Combined false alert rates of blanks and distractors from the scenarios and standards, excluding the ORTs, across all three trials.

Figure 2 compares the positive and false alert rates for both the scenarios and standards. The ORTs were removed from the standard calculations to simplify the data analysis. Across all three trials, Trial 2 had the highest false alert rate for both real-world scenarios and Standard 092 searches at 15 and 13%, respectively, while also having the highest correct response rates. Figure 3 further delineates the types of false responses that occurred during the trials. The false alerts were categorized by blanks, such as if the canine alerted to empty TADDs and/or empty boxes, and distractors, such as those listed in Tables 1, 2. Canines alerted to distractors more frequently in both the scenarios (14%) and standard (8%) than blanks at 8 and 7%, respectively. Canines had the highest false alerts on Sharpies and anti-static bags. There were a few additional false alerts that were neither on distractors nor blanks; these were classified as unknown false alerts.

### 3.2 Individual team performance

**Figure 4:**
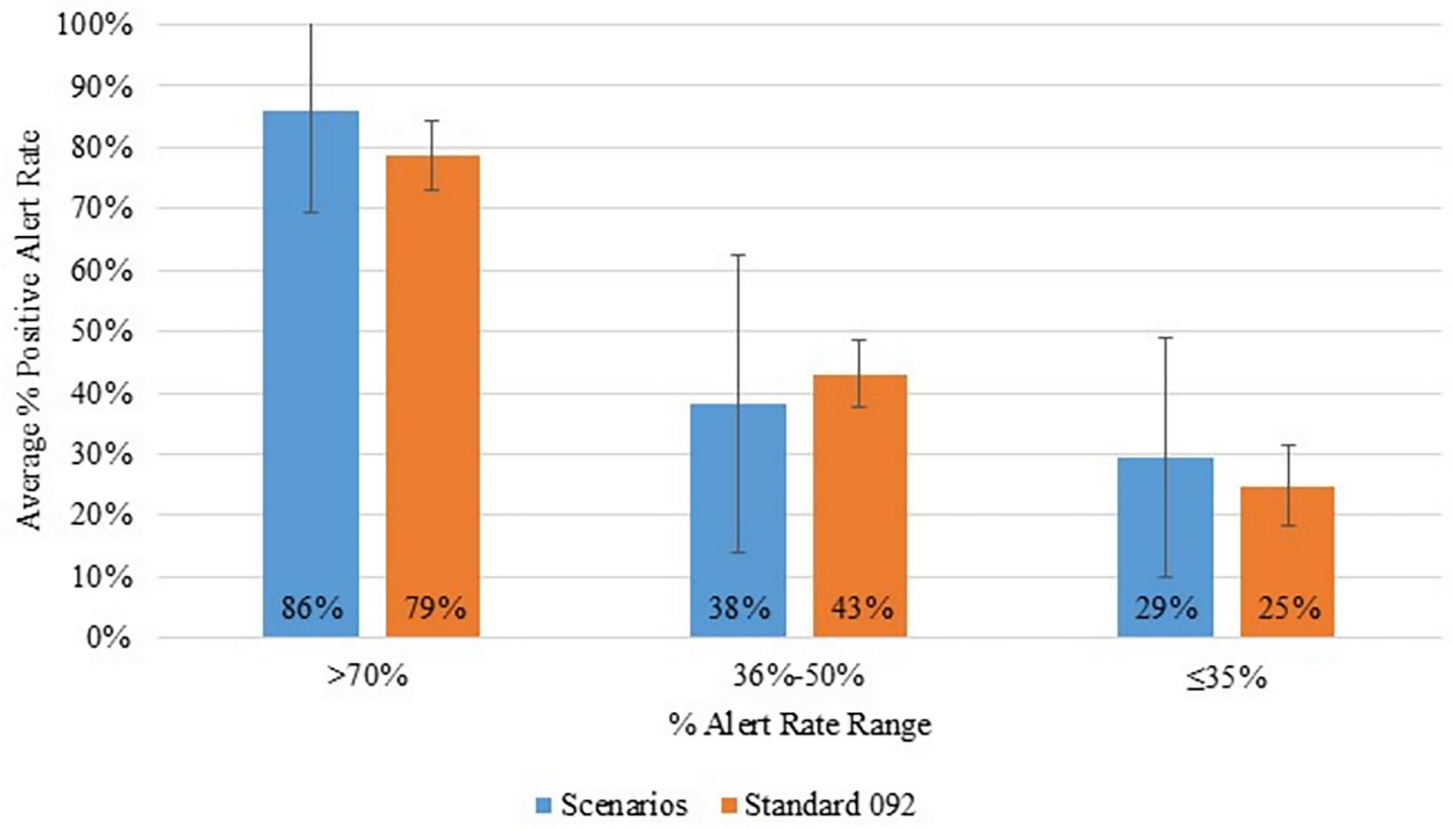
Average percent positive alert rates for all teams that had positive alert rates above 70%, between 36 and 50%, and below 35% on Standard 092 and completed at least one full day of the trial. The average percent positive alert rates were added for the scenarios for comparison.

Analyzing the individual canine/handler team responses (Supplementary information) revealed that no team would have passed the OSAC certification. Two teams were excluded from the data set as they had only completed the morning session of day one of the trial. Standard 092 requires a 90% correct alert rate with a less than 10% false alert rate for a successful certification. Five teams achieved a 70% or greater positive alert rate and a less than 10% false alert rate. An additional three teams achieved 70% or greater positive alert rate, but a false alert rate greater than 10%. The average percent alert rate of this high-achieving group was 79% ± 6% on Standard 092 and 86% ± 16% for the scenarios with 4 out of the 8 teams achieving a 100% positive alert rate on the scenarios (Figure 4), indicating high success on the Standard 092 assessment was correlated to high success on the real-world scenarios. Low-achieving groups were also examined. Twenty-one (21) teams fell into the range of 36 and 50% performance rate, and 15 teams performed less than 35% on the Standard 092 assessments (Figure 4). While the performance of the teams in the 36–50% group on the scenarios was more variable, overall, the performance of the low-achieving groups on the Standard 092 also appeared correlated to low success on the real-world scenarios.

### 3.3 Results based on explosive type

**Figure 5:**
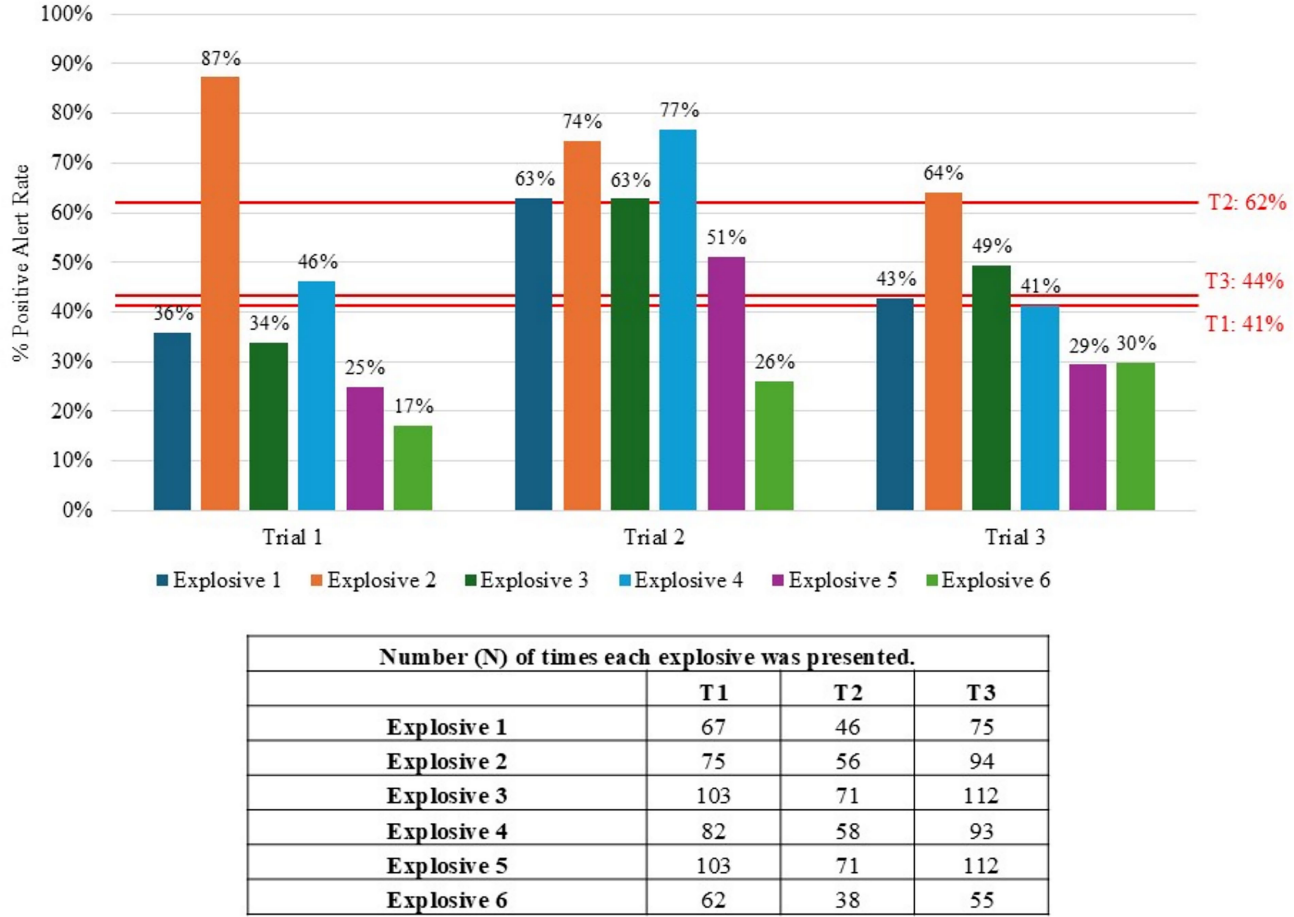
Overall detection rates based on explosive type used in Standard 092 portion for the three trials. The red lines represent the average percent positive rates from each trial.

Six different explosives were used throughout the study. Figure 5 provides information on canine/handler team performance based on explosives used in the Standard 092 portion of the trial. Overall, Explosive 2 had the highest positive response across the three trials at 87, 74, and 64%. In addition to Explosive 2, teams from Trial 1 performed above their overall percent positive alert rate on Standard 092 (shown by the red line in Figure 5) on Explosive 4, teams from Trial 2 on Explosives 1, 3, and 4, and teams from Trial 3 on Explosive 3. Explosive 6 had the lowest detection rates across all three trials, and Explosive 5 had low detection rates on Trials 1 and 3.

**Figure 6:**
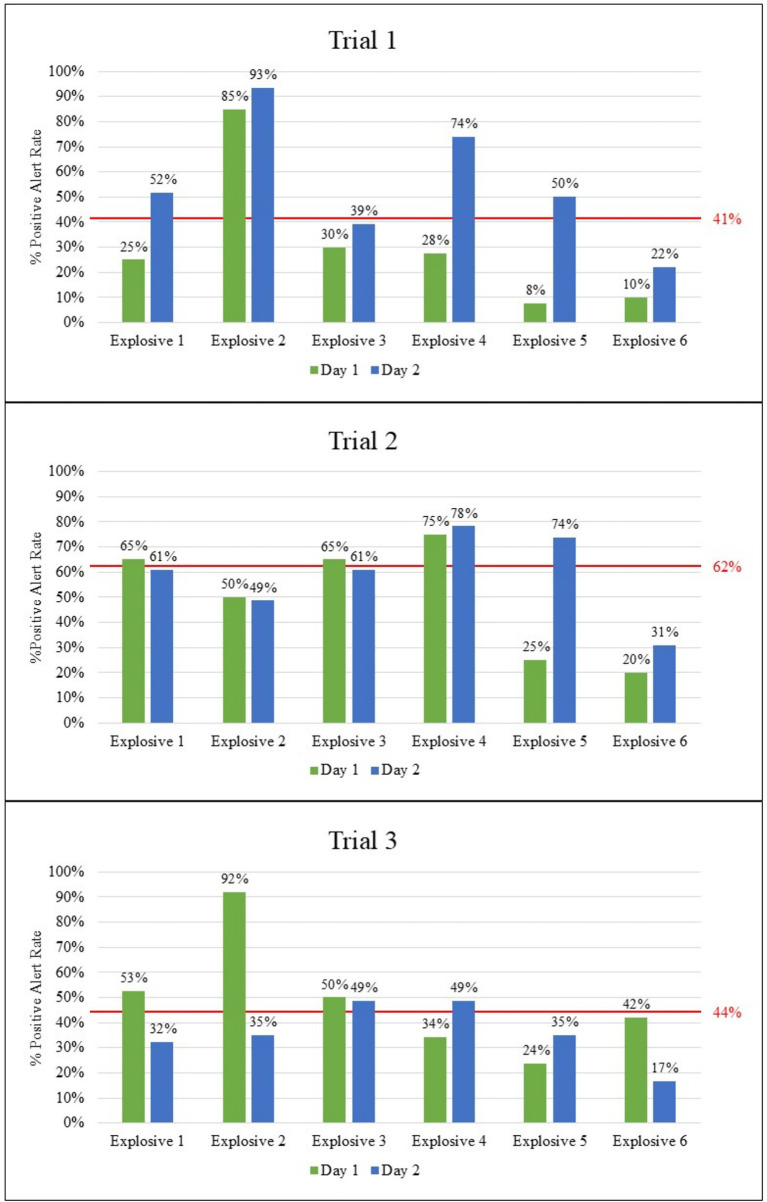
Comparison of positive response rates between Day 1 and Day 2 of all three trials separated by explosive type.

Figure 6 presents a comparison of performance between the first and second day of the trial, categorized by explosive type. Generally, a higher positive alert rate was observed on the second day of the trial for most of the explosives in Trials 1 and either an increase or consistent response in Trial 2; however, Trial 3 rates were more varied. The most notable improvement of detection was on Explosive 4 and 5 in Trial 1 with an increase of 28 to 74% alert rate and 8 to 50% alert rate, respectively, and on Explosive 5 in Trial 2 with an increase of 25 to 74% alert rate, implying that exposure to the target improved later detection.

Interestingly, the opposite trend was observed in Trial 3, where overall detection performance either declined or remained unchanged across explosive types, most notably for Explosive 2, which showed a decrease in alert rate from 92% on the first day to 35% on the second.

## 4 Discussion

### 4.1 Overall performance of canine/handler teams on standard 092 assessments

The goal of this proof-of-concept study the performance of working canine/handler teams on certification tests developed in alignment with the OSAC explosive detection Standard 092, as well as their performance on real-world scenarios designed to mimic operational conditions, utilizing 56 teams from three locations in the U.S. Canine/handler team performance varied greatly between the three trials. Trial 2 had the overall highest rate of true positives for both Standard 092 and the real-world scenarios. Trials 1 and 3 yielded similar, but significantly lower, true positive rates than seen in Trial 2 for Standard 092 assessments. Canine/handler teams who participated in Trial 3 seemed to struggle with the real-world scenarios more than the Standard 092 assessments, in contrast to observations in Trials 1 and 2. True positive rates also varied based on which of six different explosives was presented, suggesting that teams may not have had access to some of the explosives as often during training. Many canine/handler teams showed better performance on Day 2 of the trial, with teams in Trial 1 showing the greatest improvement from Day 1 to Day 2. In contrast, Trial 3 teams showed either no improvement or worse detection rates on Day 2 compared to Day 1.

When evaluating performance at the individual team level, none of the 54 teams that participated in at least one full day of a trial met the performance threshold required for certification under Standard 092. The standard requires a correct response rate of 90% with a false alert rate of less than 10%. Team 9 from Trial 2 was the highest scoring team with an 88% correct alert rate, with a 6% false alert rate on Standard 092 assessments. Seven other teams from across the three trials achieved a correct alert rate greater than 70%, while the remaining 46 teams fell below this passing threshold.

Several factors may account for the failure to meet certification requirements. One possibility is that the OSAC standard represents a particularly stringent and demanding certification process. Even teams accustomed to passing other certification tests may have struggled under the higher demands of the OSAC protocol. In comparison, other certification protocols have been cited as less rigorous than the OSAC standards for excluding the ORT (22), having smaller search areas for rooms and vehicles (22, 23), and having ill-defined requirements for the certification, leaving it up to the interpretation of the evaluator (24). Due to the length of testing, canines may have experienced fatigue or frustration which may have contributed to poor performance. This could have been especially true during prolonged or difficult searches such as the ORT or the vehicle search.

Second, four of the six explosives used within the trial, and required by Standard 092, can be difficult or expensive to obtain. Teams that showed reduced efficacy with these particular explosive materials likely had limited exposure to them during routine training. Indeed, four of the six explosives used in these trials can be very challenging to access. Thus, teams that performed fairly well overall but struggled on trials with particular explosives likely had less exposure to these materials. In many cases, these teams may only encounter these explosives once or twice a year when completing their certification hosting by other agencies, such as the North American Police Working Dog Association (NAPWDA) (25) and the National Police Canine Association (NPCA) (26). In order to increase proficiency, a team should train with multiple exemplars of target odors to improve generalization. If teams are only exposed to one source of a target odor, it could lead to discrimination, leading to no alert on a target of a similar make-up. To improve generalization, it is recommended that EDD teams be exposed and trained to a variety of training aids and training samples to promote generalization (2, 27).

Finally, teams may have experienced performance anxiety or felt unfamiliar with the trial as a whole, leading to less than optimal performance, especially on the first day. This interpretation is supported by the pattern of improved detection rates on Day 2 (see Figure 6), as this may reflect increased comfort with the flow of the trial and reduced anxiety. Additionally, performance differences could have also resulted from positive contingency and reinforcement history.

We also found differences in false alert rates across the three trials. Teams in Trial 1 achieved a low false alert rate on the Standard 092 tests (6%) and on the real-world scenarios (7%). In contrast, Trial 2 yielded the highest false alert rates for Standard 092 tests (13%) and Real-World Scenarios (15%), higher than the maximum false alert rate required to pass the OSAC certification (10%). Other certification programs, such as those offered by NAPWDA, require a comparable level of performance, mandating a minimum accuracy rate of 91.6% and permitting only a single miss over the course of the certification evaluation (25, 28).

Given that Trial 2 also yielded the highest overall correct alert rate, these patterns may reflect a response bias rather than a difference in discriminability. Trial 1 teams may be more conservative in calling alerts, resulting in fewer false alerts in addition to fewer correct alerts, while canine/handler teams in Trial 2 tended to be more liberal in their alert calls. Thus, Trial 2 yields higher false alert and correct alert rates. Teams from Trial 3 seemed to struggle with discriminating between target odors and distractors, though, because their correct and false alert rates were more similar than seen in the other trials.

In the context of explosives detection, these response tendencies may reflect strategic real-world tradeoffs. Being more willing to call an alert could be preferable, even if it means there will be more false alarms. A team that is too conservative with calling alerts may risk clearing an area when an explosive is merely very well hidden or unusual in some way.

### 4.2 Comparison of performance of standard 092 to scenarios

Another objective of this study was to determine if the observed performance rate on Standard 092 could predict how well canines would perform in operational contexts. Standard 092 recommends incorporating searches reflecting circumstances that canine/handler teams may face operationally into both training and certification. A key limitation of standardized search assessment scenarios is that, while they offer a controlled and consistent environment conducive to fair and reproducible certification, they lack the complexity and unpredictability of real-world operational settings. Real operational searches are typically more challenging (due to various distractions and attempts to mask or conceal the target) and unpredictable.

Overall, if teams performed well on Standard 092, they also tended to perform similarly on the real-world scenarios (Figure 1). The individual team data reinforced this finding as the teams who performed best on the Standard 092 also performed well when completing the Real-World Scenarios. Likewise, teams that did not perform well on Standard 092 also did not perform well on the real-world scenarios (Supplementary Tables 4, 5). There were only two teams that were outliers, achieving a correct alert rate of 36 to 50% on Standard 092 tests, but 100 and 80% on the Real-World Scenarios. This suggests that meeting the Standard 092 criteria may predict success in operational contexts too.

### 4.3 False alerts

Distractors (non-target, intentionally placed items) made up the highest percentage of false alerts across all three trials (9%). The highest false alert rate was on Sharpie markers. Other distractors odors that had high false alert, included anti-static bags, dryer sheets, isopropanol wipes, rubber bands, Tang, latex gloves, Play-Doh, banana peels, motor oil, and taco seasoning. Many of these are commonly used when training canines. Sharpie markers are typically used to indicate the contents inside a container or training aid, while anti-static bags are used to store explosives to protect against static electricity. Dryer sheets are reportedly used to decrease the static on canines before searching, and isopropanol wipes are used for cleaning and disinfecting items or an area before and after placing odors to minimize the chance of cross-contamination. Empty TADDs yielded the highest false alert rate of the blank items. This could have been a visual cue, as sometimes the canines were able to dislodge hidden distractors. The TADDs may also have an associated odor to which the canines tend to respond if these devices are regularly used during training. False alert rates of this type can be mitigated through regular training and certifications using distractors and matched blanks.

### 4.4 Parcel search

**Figure 7:**
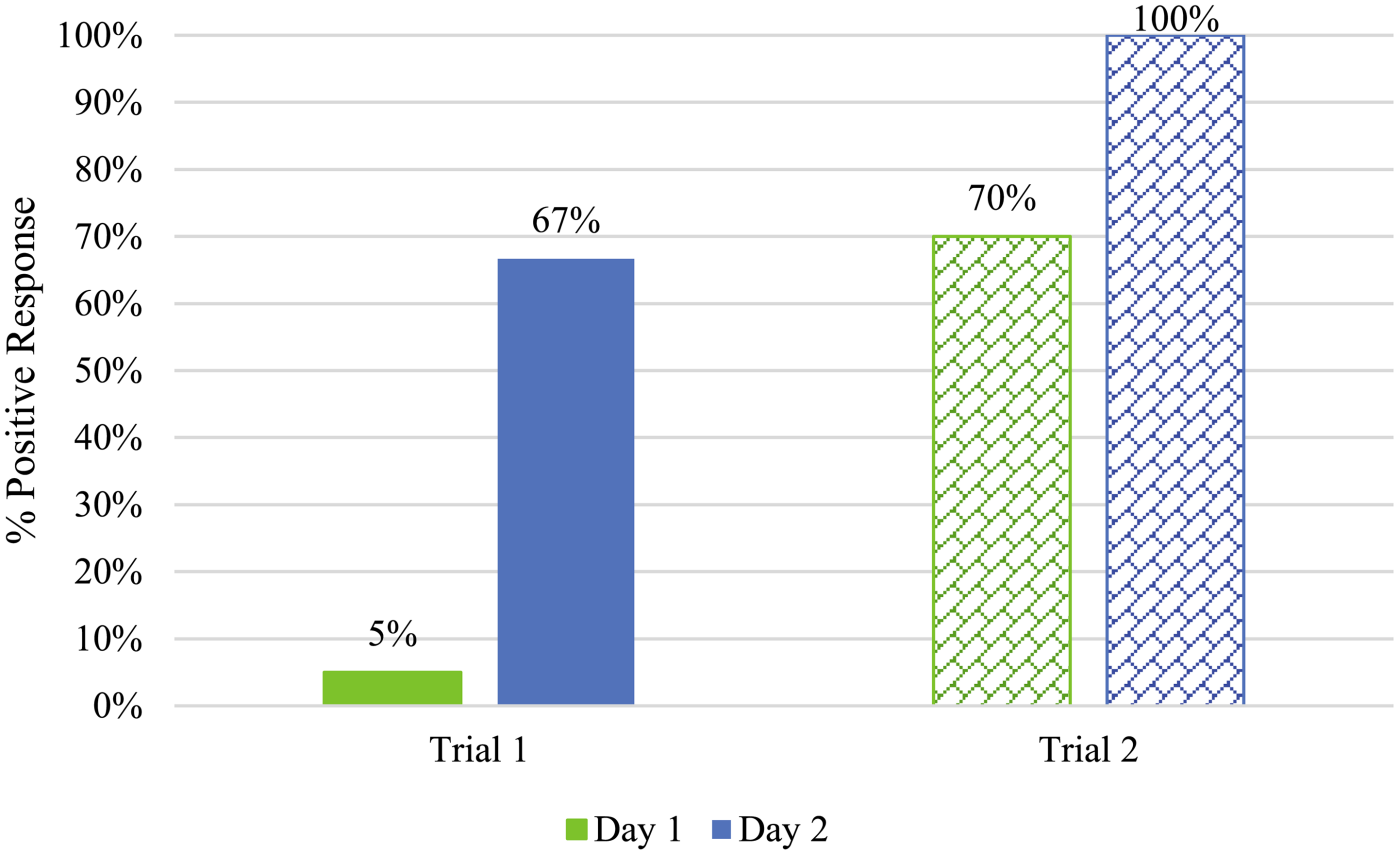
Comparison of parcel search data of Explosive 4 on Days 1 and 2 of Trials 1 and 2. Solid fill indicated a box was closed with tape (Trial 1) while a patterned fill indicated a box closed in a crosswise fashion with no tape (Trial 2). Trial 1, Day 1 (*N* = 20), Trial 1, Day 2 (*N* = 12), and Trial 2, Days 1 and 2 (*N* = 10).

One assessment that was particularly challenging for canine/handler teams was the parcel searches, where teams had difficulty locating the two explosive hides in a ten-box line-up, as delineated in the Standard 092 assessments. In Trial 1, parcels were filled with packing material and office supplies and taped closed (a single line of packing tape across the middle seam on top and bottom). Many handlers attributed the difficulty to the fact that they did not normally train or certify with “closed” boxes. In Trial 2, the boxes contained only the targets or distractors, with no additional packing material added, and were closed in a crosswise fashion with no tape involved. Figure 7 provides the results of Days 1 and 2 of the parcel searches for Explosive 4, as this explosive was used in both of the parcel searches. On the first day of Trial 1, teams correctly identified the box containing Explosive 4 only 5% of the time; however, the detection rate greatly improved on Day 2 to 67% detection rate. Comparatively, Trial 2 similarly had a detection rate of 70% on Day and 100% on Day 2.

As one would expect, the canines had a more challenging time finding the target odor when the boxes were taped closed than when they had just been crossed over. This shows an underlying problem with the way some teams train for parcel searches. It is unlikely that an explosive being concealed within a parcel would remain unsealed, as the perpetrator would want to hide the energetic material. Studies have shown that packaging materials, such as cardboard, limit the amount of vapor that escapes into the open environment (29), making it harder to detect. However, other studies have shown that trained detection canines are highly efficient and effective at locating contained targets with proper training (30). The data here also suggests that the canine/handler teams were capable of completing this task after some practice (see Day 2 data in Figure 7). This reinforces the idea that materials used in training and certification should be prepared in ways that prepare canines for operational contexts.

### 4.5 Study limitations

The paradigm provided by the OSAC standard was challenging to carry out, both in regards to time and space. The certification took place over two full days starting around 8:30 am and ending around 5 pm. With the real-world scenarios removed off the itinerary, the days would shorten, but not by much. Finding a facility to accommodate each individual search where no overlapping of rooms was difficult. In the first trial, multiple rooms needed to be combined to meet the size requirements of the room searches and the same rooms needed to be reused on the second day just due to lack of extra space within the facility. For agencies that either have or can work with an organization that has such large facilities, the certification may be feasible to achieve, but for smaller organizations that do not have access to a facility it may be more challenging. Additionally, since the study was not double-blinded, it was possible that assessors provided unintentional clues to handler about the location of targets. This would lead to the handler providing unintentional clues cues that influenced canine performance, potentially skewing the results.

Although our study did not directly measure cognitive or biological state variables, several factors could have plausibly contributed to detection failures. Mostly commonly, training issues, such as inferior training materilas or limited training opportunities for either the handler or canine, or sufficient searching patterns [31], can be pinpointed for detioration in team performance. However other complications, may have been at play. For example, the temperature was high in most days of all three trials (Day 2 of the SE trial was the exception). Several searches were outdoors, which, in addition to the walking from the vehicle kenneling location to the indoor searchs, could cause excessive exertion, though the canines were given the opportunity to rest whenever needed. It has been shown that increased physical exertion can degrade olfactory sensitivity, where dogs on a treadmill exhibited a drop in accuracy rate from approximately 87% to below 45% for weak odor concentrations after moderate-intensity exercise over time [32]. Similarly, intrinsic characteristics such as arousal and motivation—often described as ‘hunt drive’ or ‘search arousal’—have been linked to detection success across operational contexts [33, 34]; however, while high arousal may initially elevate engagement, excessive arousal, such as marked excitability, can impair detection accuracy, potentially via panting interfering with olfactory sampling [35, 36]. Non-cognitive variables like handler stress due to unknown or testing scenarios can also undermine performance, regardless of a dog’s detection capability [31]. Although these factors were not directly measured in our study, considering their potential influence provides a richer interpretation of the observed protocol differences and highlights valuable directions for future investigation.

## 5 Conclusion

The study aimed to objectively assess the OSAC explosive detection Standard 092 and evaluate the performance of EDD teams in the United States. The results of the Std 092 portion of the trials showed a low success rate across all trials, with no participating canine team successfully passing the certification requirements as delineated by the standard benchmark. There are several potential reasons that so many teams struggled with the Standard 092 tests, but the results did reveal a need for better standardization in the training of explosive detection dog teams. Some canine/handler teams were also challenged by explosives they did not have frequent access to for training. Improved correct alert rates for these explosives on Day 2 of some of the trials demonstrated the importance of regular training with common explosives, even if they are difficult to access. Additionally, it is recommended that boxes used for parcel searches in both training and certification assessments should be closed well with packaging tape to simulate real-life searches, given that many teams incurred issues with closed parcels within these trials.

Finally, the data showed that proficiency on the Standard 092 certification assessment can reflect operational performance, as detection rates between the Standard 092 assessments and the real-world scenarios were comparable, indicating that the searches required by Standard 092 may be a good reflection of what canine/handler teams experience during operational searches. Future studies should expand to include trials in geographic locations in the northern regions of the United States to better understand the variability in canine/handler team performance on a national level. Further studies of this type are also important to establish the efficacy, feasibility, and predictive power of other standardization certification for other detections domains, such as illicit substances and human remains, to continue to strengthen the empirical foundation of canine detection in forensic settings.

## Data Availability

The original contributions presented in the study are included in the article/Supplementary material, further inquiries can be directed to the corresponding authors.
