# Supplementary material for: Explosive detection canines in the field: a multi-site black box validation study
Source: Front Vet Sci. 2025 Oct 16;12:1668317. doi: 10.3389/fvets.2025.1668317 (PMC12571827; doi:10.3389/fvets.2025.1668317)
Supplement: Supplementary file 1 [file Supplementary_file_1.docx]

Supplemental Material File 1: Participation of all canine teams that participated in the study.

Supplemental Table 1: Participation record for all canine teams that participated within the trial. An X represents a team that participated in the scenario or standard. A grayed out cell represents a canine team that did not participate in the scenario or standard.


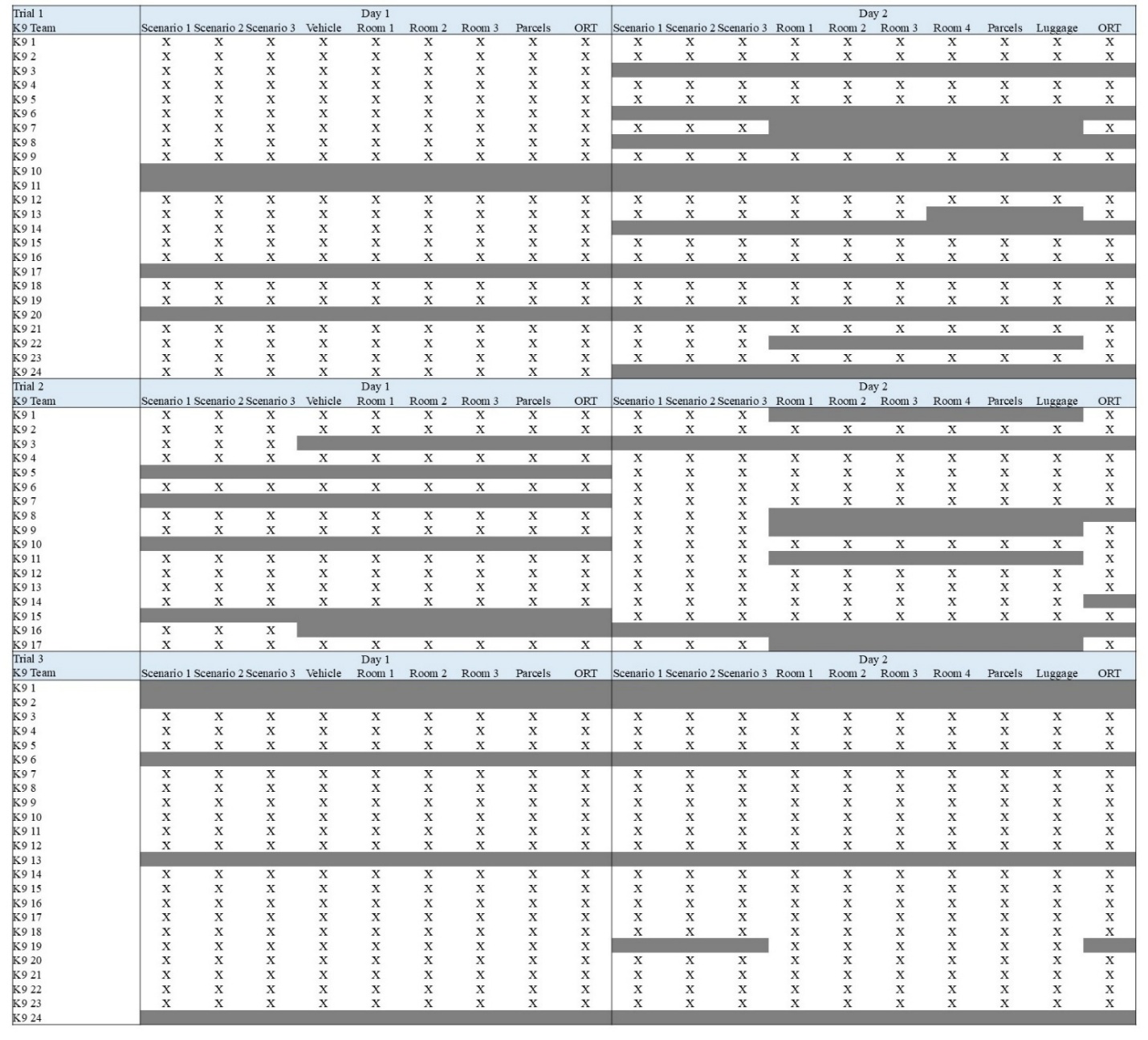


Supplemental Material File 1: General information collected by a survey from handlers who participated in the study.

Supplemental Table 2: Canine team general information. Collection of general information started during Trial 2, so not all information is available for participants in Trial 1.

| Team | cANINE BREED | Canine age (year) | Dog in service (years) | Handler in service (years) | Most recent certification (year) |
| --- | --- | --- | --- | --- | --- |
| 1 | German Shepherd | 7 | 4 | 4 | 2024 |
| 2 | German Shepherd | 3 | 1 | 1 | 2024 |
| 3 | Malinois | 3 | 1 | 7 | 2024 |
| 4 | German Shepherd | 5 | 3 | 3 | 2024 |
| 5 | Malinois | 7 | NA (3) | NA (3) | NA |
| 6 | Malinois | 5 | NA | NA | NA |
| 7 | Malinois | 5 | NA | NA | NA |
| 8 | German Shepherd | 6 | NA | NA | NA |
| 9 | German Shepherd | 6 | 5 | 5 | 2024 |
| 12 | Malinois | 5 | NA | NA | NA |
| 13 | German Shepherd | 4 | 2 | 17 | 2024 |
| 14 | NA | NA | NA | NA | NA |
| 15 | German Shepherd | 5 | NA | NA | NA |
| 16 | Dutch Shepherd | 8 | NA | NA | NA |
| 18 | Malinois | 5 | NA | NA | NA |
| 19 | Pointer | 4 | NA | NA | NA |
| 21 | Labrador | 5 | 3 | 3 | 2024 |
| 22 | Labrador | 7 | NA | NA | NA |
| 23 | German Shepherd | 4 | NA | NA | NA |
| 24 | German Shepherd | 2 | NA | NA | NA |
| Team | Canine Breed | Canine age (year) | Dog in service (years) | Handler in service (years) | Most recent certification (year) |
| 1 | Golden Retriever | 4 | 3 | 3 | 2024 |
| 2 | Malinois | 5 | 0 | 0 | N/A |
| 3 | Labrador | 6 | 5 | 7 | 2024 |
| 4 | German Shepherd | 6 | 3 | 3 | 2024 |
| 5 | Belgian Malinois | 4 | 3 | 8 | 2024 |
| 6 | Labrador | 5 | 4 | 4 | 2024 |
| 7 | Labrador Retriever | 7.5 | 2 | 10 | 2024 |
| 8 | German Shepherd | 9 | 9 | 8 | 2024 |
| 9 | German Shepherd | 10 | 7 | 6 | 2022 |
| 10 | German Shorthair Pointer | 5 | 3 | 3 | 2024 |
| 11 | Belgian Malinois/ German Shepherd | 4 | 2.5 | 3 | 2024 |
| 12 | Labrador | 5 | 2 | 6 | 2024 |
| 13 | Belgian Malinois/ German Shepherd | 5 | 3.5 | 3.5 | 2024 |
| 14 | Belgian Malinois | 3.5 | 2.5 | 21 | 2024 |
| 15 | Labrador | 5 | 3 | 3 | 2024 |
| 16 | German Shorthair Pointer/ Labrador | 2.5 | 2 | 2 | 2024 |
| 17 | Malinois | 2.5 | NA | NA | 2024 |
| Team | Canine Breed | Canine age (year) | Dog in service (years) | Handler in service (years) | Most recent certification (year) |
| 1 | German Shepherd | 9 | 2 | 6 | 2025 |
| 2 | Belgian Malinois | 2 | <1 | <1 | 2025 |
| 3 | Labrador | 4 | 3 | 3 | 2025 |
| 4 | NA | 6 | 2 | 3 | 2023 |
| 5 | German Shorthair Pointer | 8.5 | 7 | 7 | 2024 |
| 6 | Belgian Malinois | 5 | 3 | 8 | 2024 |
| 7 | Labrador | 4 | 3 | 3 | 2024 |
| 8 | Labrador Retriever | 3 | 1 | 13 | 2024 |
| 9 | NA | 6 | 3.5 | 3.5 | 2025 |
| 10 | German Shorthair Pointer | 3 | 1 | 3 | 2024 |
| 11 | German Shorthair Pointer | 6 | 4 | 3 | 2025 |
| 12 | Dutch Shepherd | 4.5 | 3 | 9+ | 2025 |
| 13 | German Shepherd | 3 | 2 | 2 | 2025 |
| 14 | Malinois/ German Shepherd | 5 | 4 | 4 | 2025 |
| 15 | Labrador Retriever | 2 | 1 | 14 | 2025 |
| 16 | German Shepherd | 3 | 1.5 | 1.5 | 2025 |
| 17 | Labrador | 4 | 3 | 3 | 2024 |
| 18 | English Labrador | 4 | 2 | 2 | 2024 |
| 19 | Labrador/ Pointer | 3 | 2 | 2 | 2025 |

Supplemental Material File 3: More in-depth information about the canine trials set-ups:

Standard Assessment – The information here was gathered from Standard 092, found within the Reference section.

- Odor recognition test
  - Target odors were placed in a manner where the odor was readily available but are not visible to the canine and handler. A minimum of 18 sample containers shall be used, and these containers must be spaced out a minimum of 3 ft apart. For this study, 24 sample containers were used. The sample containers were placed in a manner that minimized environmental influences that may affect the odor. The containers used were clean, unused and identical to each other and had perforations that allowed odor to be readily available. For this experiment, carboard boxes from Uline were used, but containers could be metal paint cans, ointment tins, etc. A minimum of six target odors and six distractor odors were placed randomly among the sample containers. The other 12 sample containers contained blank TADDs or were left completely empty. Teams were allowed to search each sample container twice and have a maximum of 5 minutes to complete the search.
- Baggage/ Parcel Operational Assessment
  - A minimum of 10 baggage/parcels items shall be included within the operational line-up. Baggage/ parcel options included hard and soft-shelled suitcases and carboard boxes. Other baggage/parcel options include clothing bags, briefcases, carboard boxes, envelopes, etc. A minimum of two baggage/parcels items contained a target odor. These items were randomly place and were not placed next to each other. Baggage/parcels were spaced out a minimum of 3 ft from each other to allow canine/handler teams to move around the items. Canine/handler teams were permitted 3 min to search the area.
- Room Operational Assessment
  - A minimum of three rooms shall be used for the assessment. For the purpose of this study, four rooms were used. The rooms should be between 200 ft^2^ (20 m^2^) to 1200 ft^2^ (100 m^2^) containing items such as furniture, shelves, distractors, etc. If the room exceeded the maximum size, it was sectioned off to meet the size requirement. Only one target was placed in a single room and was placed anywhere from ground level to 6 ft (2 m) high. One out of the four rooms were kept blank, meaning no target odor was present. The canine/handler team was only allowed two passes per room and should take no more than 1.5 min per 100 ft^2^.
- Vehicle Assessment
  - A minimum of 10 vehicles shall be used for the assessment. A maximum of one target placed per 5 vehicles and only one target shall be placed in a single vehicle. Target odors can be placed either in the exterior or the interior of the vehicles. Vehicles containing targets should not be parked next to each other. For this study, 10 vehicles were used and only one target was present within the line-up. Cars were spaced far apart enough to provide enough room for canine/handler teams to move in between vehicles. The assessment should take no more than 2 min per vehicle, meaning if 10 cars are used, the teams are allotted 20 minutes to complete the search.

Reference:

1. *Standard for Training and Certification of Canine Detection of Explosives | American Academy of Forensic Sciences*. https://www.aafs.org/asb-standard/standard-training-and-certification-canine-detection-explosives (accessed 2025-06-05)

Supplemental Material File 4: Individual team results from the scenarios and Standard 092.

Supplemental Table 3: Individual team results for both the scenarios and Standard 092 of canine/handler teams that had % positive alert rates > 70% during the Standard 092 portion of the trials. Canine/handler teams in blue participated in Trial 1, canine/handler teams in red participated in Trial 2, and canine/handler teams in black participated in Trial 3.

| **K9 Team** | **Outcome** | **Hit** | **Target Present** | **Percent Correct Alert Rate** | **False Alert** | **Distractors and Blanks Present** | **Percent False Alert Rate** |
| --- | --- | --- | --- | --- | --- | --- | --- |
| 9 | Scenario | 5 | 5 | 100% | 0 | 22 | 0% |
|  | Standard 092 | 15 | 17 | 88% | 4 | 66 | 6% |
| 7 | Scenario | 2 | 3 | 67% | 0 | 8 | 0% |
|  | Standard 092 | 9 | 11 | 82% | 4 | 35 | 11% |
| 13 | Scenario | 5 | 5 | 100% | 3 | 22 | 14% |
|  | Standard 092 | 18 | 22 | 82% | 12 | 101 | 20% |
| 14 | Scenario | 4 | 5 | 80% | 5 | 22 | 23% |
|  | Standard 092 | 13 | 16 | 81% | 5 | 83 | 6% |
| 11 | Scenario | 3 | 5 | 60% | 4 | 22 | 18% |
|  | Standard 092 | 17 | 22 | 77% | 10 | 98 | 10% |
| 5 | Scenario | 3 | 3 | 100% | 0 | 8 | 0% |
|  | Standard 092 | 8 | 11 | 73% | 3 | 35 | 9% |
| 15 | Scenario | 3 | 3 | 100% | 0 | 8 | 0% |
|  | Standard 092 | 8 | 11 | 73% | 7 | 35 | 20% |
| 21 | Scenario | 4 | 5 | 80% | 2 | 23 | 4% |
|  | Standard 092 | 16 | 22 | 73% | 4 | 99 | 7% |

Supplemental Table 4: Individual team results for both the scenarios and Standard 092 of canine/handler teams that had % positive alert rates between 36%-50% during the Standard 092 portion of the trials. Canine/handler teams in blue participated in Trial 1, canine/handler teams in red participated in Trial 2, and canine/handler teams in black participated in Trial 3.

| **K9 Team** | **Outcome** | **Hit** | **Target Present** | **Percent Correct Alert Rate** | **False Alert** | **Distractors and Blanks Present** | **Percent False Alert Rate** |
| --- | --- | --- | --- | --- | --- | --- | --- |
| 20 | Scenario | 2 | 5 | 40% | 1 | 22 | 5% |
|  | Standard 092 | 11 | 22 | 50% | 8 | 98 | 8% |
| 21 | Scenario | 0 | 5 | 0% | 3 | 22 | 14% |
|  | Standard 092 | 11 | 22 | 50% | 8 | 98 | 8% |
| 22 | Scenario | 2 | 5 | 40% | 5 | 22 | 23% |
|  | Standard 092 | 11 | 22 | 50% | 10 | 98 | 10% |
| 4 | Scenario | 1 | 5 | 20% | 6 | 22 | 27% |
|  | Standard 092 | 11 | 22 | 50% | 15 | 98 | 15% |
| 6 | Scenario | 2 | 5 | 40% | 4 | 22 | 18% |
|  | Standard 092 | 6 | 12 | 50% | 12 | 44 | 27% |
| 22 | Scenario | 1 | 5 | 20% | 2 | 23 | 9% |
|  | Standard 092 | 8 | 17 | 47% | 4 | 99 | 4% |
| 7 | Scenario | 1 | 5 | 20% | 6 | 22 | 27% |
|  | Standard 092 | 10 | 22 | 45% | 11 | 98 | 11% |
| 23 | Scenario | 0 | 5 | 0% | 4 | 22 | 18% |
|  | Standard 092 | 10 | 22 | 45% | 12 | 98 | 12% |
| 9 | Scenario | 2 | 5 | 40% | 4 | 22 | 18% |
|  | Standard 092 | 10 | 22 | 45% | 13 | 98 | 13% |
| 10 | Scenario | 5 | 5 | 100% | 2 | 8 | 25% |
|  | Standard 092 | 5 | 11 | 45% | 6 | 35 | 17% |
| 19 | Scenario | 1 | 2 | 50% | 2 | 14 | 14% |
|  | Standard 092 | 7 | 16 | 44% | 6 | 80 | 8% |
| 5 | Scenario | 1 | 5 | 20% | 4 | 23 | 17% |
|  | Standard 092 | 9 | 22 | 41% | 3 | 99 | 3% |
| 10 | Scenario | 3 | 5 | 60% | 0 | 22 | 0% |
|  | Standard 092 | 9 | 22 | 41% | 6 | 98 | 6% |
| 14 | Scenario | 2 | 5 | 40% | 2 | 22 | 9% |
|  | Standard 092 | 9 | 22 | 41% | 12 | 98 | 12% |
| 1 | Scenario | 1 | 5 | 20% | 8 | 22 | 36% |
|  | Standard 092 | 7 | 17 | 41% | 7 | 66 | 11% |
| 18 | Scenario | 1 | 5 | 20% | 4 | 22 | 18% |
|  | Standard 092 | 8 | 22 | 36% | 5 | 98 | 5% |
| 9 | Scenario | 3 | 5 | 60% | 2 | 23 | 9% |
|  | Standard 092 | 8 | 22 | 36% | 3 | 99 | 3% |
| 16 | Scenario | 2 | 5 | 40% | 1 | 23 | 4% |
|  | Standard 092 | 8 | 22 | 36% | 4 | 99 | 4% |
| 3 | Scenario | 1 | 2 | 50% | 1 | 14 | 7% |
|  | Standard 092 | 4 | 11 | 36% | 3 | 47 | 6% |
| 15 | Scenario | 4 | 5 | 80% | 2 | 23 | 9% |
|  | Standard 092 | 8 | 22 | 36% | 7 | 99 | 7% |
| 12 | Scenario | 2 | 5 | 40% | 3 | 22 | 14% |
|  | Standard 092 | 8 | 22 | 36% | 11 | 98 | 11% |

Supplemental Table 5: Individual team results for both the scenarios and Standard 092 of canine/handler teams that had % positive alert rates ≤ 35% during the Standard 092 portion of the trials. Canine/handler teams in blue participated in Trial 1, canine/handler teams in red participated in Trial 2, and canine/handler teams in black participated in Trial 3.

| **K9 Team** | **Outcome** | **Hit** | **Target Present** | **Percent Correct Alert Rate** | **False Alert** | **Distractors and Blanks Present** | **Percent False Alert Rate** |
| --- | --- | --- | --- | --- | --- | --- | --- |
| 11 | Scenario | 2 | 5 | 40% | 3 | 22 | 14% |
|  | Standard 092 | 6 | 17 | 35% | 8 | 66 | 12% |
| 18 | Scenario | 2 | 5 | 40% | 1 | 23 | 4% |
|  | Standard 092 | 7 | 22 | 32% | 5 | 99 | 5% |
| 8 | Scenario | 2 | 5 | 40% | 0 | 22 | 0% |
|  | Standard 092 | 7 | 22 | 32% | 6 | 98 | 6% |
| 7 | Scenario | 3 | 5 | 60% | 3 | 23 | 13% |
|  | Standard 092 | 5 | 17 | 29% | 5 | 65 | 8% |
| 12 | Scenario | 2 | 5 | 40% | 2 | 23 | 9% |
|  | Standard 092 | 6 | 22 | 27% | 3 | 99 | 3% |
| 8 | Scenario | 1 | 2 | 50% | 2 | 14 | 14% |
|  | Standard 092 | 3 | 11 | 27% | 2 | 47 | 4% |
| 19 | Scenario | 1 | 5 | 20% | 2 | 23 | 9% |
|  | Standard 092 | 6 | 22 | 27% | 6 | 99 | 6% |
| 24 | Scenario | 0 | 2 | 0% | 1 | 14 | 7% |
|  | Standard 092 | 3 | 11 | 27% | 5 | 81 | 6% |
| 5 | Scenario | 1 | 5 | 20% | 1 | 22 | 5% |
|  | Standard 092 | 6 | 22 | 27% | 16 | 98 | 16% |
| 17 | Scenario | 1 | 5 | 20% | 5 | 22 | 23% |
|  | Standard 092 | 5 | 22 | 23% | 12 | 98 | 12% |
| 14 | Scenario | 0 | 2 | 0% | 0 | 14 | 0% |
|  | Standard 092 | 2 | 11 | 18% | 1 | 47 | 2% |
| 6 | Scenario | 1 | 2 | 50% | 0 | 14 | 0% |
|  | Standard 092 | 2 | 11 | 18% | 2 | 47 | 4% |
| 23 | Scenario | 1 | 5 | 20% | 4 | 23 | 17% |
|  | Standard 092 | 4 | 22 | 18% | 5 | 99 | 5% |
| 3 | Scenario | 0 | 5 | 0% | 3 | 22 | 14% |
|  | Standard 092 | 4 | 22 | 18% | 6 | 98 | 6% |
| 17 | Scenario | 2 | 5 | 40% | 2 | 22 | 9% |
|  | Standard 092 | 2 | 17 | 12% | 2 | 66 | 3% |

Supplemental Material File 4: Supplementary Multi-Level Model Analyses

*Supplemental Table 6: Intraclass correlations (ICC) to show the variation in correct and false alerts attributable to the K9 Team, the Trial Number, and the Assessment Type. Higher ICCs indicate more variation in alerts based on the variable noted in Column 2. False alerts only include those on intentionally placed distractors or blank items.*

| **Alert Type** | **Variation attributed to…** | **ICC** | **ICC 95% CI** |
| --- | --- | --- | --- |
| Correct Alerts | K9 Team (Total = 56) | 0.10 | [0.06, 0.16] |
|  | Trial Number (1, 2, or 3) | 0.05 | [0.01, 0.67] |
|  | Assessment Type (Standard 092 Certification or Real-World Scenario) | 0.00 | [0.00, 0.01] |
| False Alerts | K9 Team | 0.02 | [0.01, 0.04] |
|  | Trial Number (1, 2, or 3) | 0.02 | [0.00, 0.46] |
|  | Assessment Type (Standard 092 Certification or Real-World Scenario) | 0.00 | [0.00, 0.10] |

Explanation of ICC values:

1. K9 Team contributed a lot of variation in correct alerts, but less variation in false alerts.
2. Trial Number contributed some variation in correct alerts and false alerts.
3. Assessment Type did not contribute much variation to these data.

*Supplementary Table 7: Multilevel Logistic Regression with* ***correct*** *alerts (1) vs. missed alerts (0) as the outcome variable, nested with K9 Team, with experience of the canine and handler, age of the canine, trial number, and trial type included as predictors. We report standardized betas (ß), p-values (p), conditional (variation explained by Level 2 and 1 predictors) and marginal R^2^ values (variation explained by Level 1 predictors) and Odds Ratios (OR).*

| **Model** | **Predictors** | ***ß*** | ***p*** | ***R^2^_conditional_*** | ***R^2^_marginal_*** | ***OR*** |
| --- | --- | --- | --- | --- | --- | --- |
| Null | None | - | .247 | 12.3% | 0.0% | - |
| Team Data 1 | Age of Canine | 0.26 | .283 | 13.1% | 0.4% | 1.08 |
| Team Data 2 | Canine – Yrs of Experience | 0.14 | .655 | 14.8% | 0.1% | 1.04 |
| Team Data 3 | Handler – Yrs of Experience | 0.26 | .422 | 14.8% | 0.4% | 1.03 |
| Trial Data 1 | Trial Type (Scenario vs. Cert) | -0.15 | .356 | 15.1% | 8.7% | 0.86 |
|  | Trial Number (2 vs. 1) | 1.13 | <.001* |  |  | 3.68 |
|  | Trial Number (3 vs. 1) | -0.07 | .756 |  |  | 0.93 |
| Trial Data 4 | Trial Type (Scenario vs. Cert) | 0.28 | .294 | 15.9% | 9.5% | 1.34 |
|  | Trial Number (2 vs. 1) | 1.31 | <.001* |  |  | 4.56 |
|  | Trial Number (3 vs. 1) | 0.18 | .505 |  |  | 1.20 |
|  | Trial Type * Number (2 vs. 1) | -0.35 | .189 |  |  | 0.56 |
|  | Trial Type * Trial Number (3 vs. 1) | -0.54 | .044* |  |  | 0.45 |

Explanation of Supplementary Table 7: These results show:

1) That Trial 2 had a significantly higher overall hit rate than Trial 1 or 3 (OR = 3.68);

2) That Trial 3 was the only trial where hit rate on the Real-World Scenarios was worse than the Certifications (OR = 0.45);

3) Hit rate did not differ significantly between Trial Type (Certification and Real-World Scenario Assessments; OR = 0.86);

3) A lot of variation in hit rates was explained by the K9 Team,

4) Trial type and number explained some variation in hit rates, but not as much as which K9 team the data were from.

*Supplementary Table 8: Multilevel Logistic Regression with* ***false*** *alerts (1) vs. correct rejections (0) as the outcome variable, nested with K9 Team, with experience of the canine and handler, age of the canine, trial number, and trial type included as predictors. We report standardized betas (ß), p-values (p), conditional (variation explained by Level 2 and 1 predictors) and marginal R^2^ values (variation explained by Level 1 predictors) and Odds Ratios (OR).*

| **Model** | **Predictors** | ***ß*** | ***p*** | ***R^2^_conditional_*** | ***R^2^_marginal_*** | ***OR*** |
| --- | --- | --- | --- | --- | --- | --- |
| Null | None | - | <.001* | 6.2% | 0.0% | - |
| Team Data 1 | Age of Canine | 0.56 | .163 | 7.6% | 0.6% | 1.09 |
| Team Data 2 | Canine – Yrs of Experience | 0.70 | .040* | 4.9% | 1.2% | 1.12 |
| Team Data 3 | Handler – Yrs of Experience | -0.08 | .840 | 5.0% | 0.0% | 1.00 |
| Trial Data 1 | Trial Type (Scenario vs. Cert) | -0.16 | .552 | 6.8% | 5.5% | 0.90 |
|  | Trial Number (2 vs. 1) | 1.90 | <.001* |  |  | 3.18 |
|  | Trial Number (3 vs. 1) | 1.00 | .007* |  |  | 1.70 |
| Trial Data 4 | Trial Type (Scenario vs. Cert) | 1.32 | .006* | 8.9% | 7.6% | 2.32 |
|  | Trial Number (2 vs. 1) | 2.42 | <.001* |  |  | 4.35 |
|  | Trial Number (3 vs. 1) | 1.62 | <.001* |  |  | 2.36 |
|  | Trial Type * Number (2 vs. 1) | -1.09 | .004* |  |  | .305 |
|  | Trial Type * Number (3 vs. 1) | -1.43 | .002* |  |  | .253 |

Explanation of Supplementary Table 8: These results show:

1) When the canine had more years of experience, this was associated with a slight increase in false alerts (OR = 1.12);

2) The teams who participated in Trial 2 were significantly more likely to false alert than teams in Trial 1 (OR = 3.18);

3) The teams who participated in Trial 3 were significantly more likely to false alert than teams in Trial 1, but less than those in Trial 2 (OR = 1.70);

4) The difference in false alert rates for Certifications versus Real-World Scenarios was significantly different in Trial 1 than seen in either Trial 2 (OR = .305) or 3 (OR = .253); and,

5) Less variation in false alerts was explained by individual teams.
